# Passive Harmonic RFID System for Buried Assets Localization

**DOI:** 10.3390/s18113635

**Published:** 2018-10-26

**Authors:** Abanob Abdelnour, Antonio Lazaro, Ramón Villarino, Darine Kaddour, Smail Tedjini, David Girbau

**Affiliations:** 1 Laboratoire de Conception et d’Intégration des Systèmes (LCIS), University Grenoble Alpes, Grenoble INP, 26000 Valence, France; abanob-assaad-amin.abdelnour@lcis.grenoble-inp.fr (A.A.); darine.kaddour@lcis.grenoble-inp.fr (D.K.); smail.tedjini@lcis.grenoble-inp.fr (S.T.); 2 Department of Electronics, Electrics and Automatic Control Engineering, Rovira i Virgili University, 43007 Tarragona, Spain; ramon.villarino@urv.cat (R.V.); david.girbau@urv.cat (D.G.)

**Keywords:** harmonic radar, RFID, underground communications, passive tag

## Abstract

A passive harmonic tag for buried assets localization is presented for utility localization. The tag design is based on a dual-polarized patch antenna at Ultra High Frequency (UHF) band. One of its feeders is connected to a frequency doubler based on a Schottky diode that generates the second harmonic, which is transmitted using a linear-polarized patch tuned at this frequency. The power received at the other feeder of the dual-polarized antenna is harvested by an RF to DC converter based on a five-stage voltage multiplier whose energy is used to bias a low-power quartz oscillator that modulates the output of the doubler. The different parts of the system are presented, and the theoretical read range is estimated as a function of the soil composition and the water content. A low-cost reader based on a software defined radio is also presented. Finally, experiments with a prototype of the tag are performed for different soil conditions.

## 1. Introduction

Pipeline transportation is considered the safest, most progressive, and most economical mode of transportation. Every day, energy resources such as refined petroleum and natural gas, as well as water supplies, are all being transported through complex underground pipeline networks from production areas or ports of entry to consumers, airports, military bases, population centers, and industry. Most pipelines are typically designed to have a lifespan of more than 25 years. However, maintenance and expansion operations are always required to meet high demands for safety, reliability, and efficiency. During the excavation process, pipeline operators face a challenging and uncertain task in accurately determining the position of buried pipelines, especially in urban areas. This leads to accidental breakage of pipes that can be very costly and causes a significant delay in civil constructions [1].

In this context, there is an increasing need to use non-destructive techniques for detection and localization of buried pipes in order to avoid and minimize failures during working processes. There are two primary methods for detecting the locations of the underground infrastructures. The first method is based on electromagnetic induction [2,3] and relies on materials being conductive or metallic, which excludes the possibility of detecting plastic pipes recently used by many pipe operators. The second method is GPR (ground penetrating radar), based on the propagation of a very short electromagnetic pulse (1–20 ns) in the frequency band of 10 MHz–2.5 GHz [4,5]. Although GPR has proven its efficiency in locating different types of pipes (especially metal pipes) and is the most widespread method, the use of this technique is quite expensive and requires experienced operators to interpret the results of complex signal processing operations.

In recent years, Radio Frequency Identification (RFID) technology has grown significantly and new applications have appeared, such as parking management based on RFID [6], tracking of objects in hospitals [7], and localization based on RFID tags [8]. However, there are also other challenges such as security aspects, interference issues [9], or undesired effects of the environment. In some of these applications, special tags for metal surfaces or embedded tags are required [5,10,11,12]. Recently, RFID systems have experienced a significant interest as a potentially cost-effective and less complicated alternative technique for marking buried items [13,14], as well as for quality monitoring of oil and gas pipelines [15]. The key challenges identified were the powering of these sensors and the communication of the data to the operator, as a result of the attenuation of the electromagnetic signals caused by the ground. To solve this problem, the use of active wireless systems [16] is a possible solution. However, as a long lifetime is required for this application, passive devices provide a more efficient solution compared with battery-powered devices. Also, passive green devices are preferred in order to avoid soil contamination due to dangerous chemical substances in battery cells. Therefore, an energy harvesting method to provide energy to the tag electronics is required. As the tags are buried, the easiest way is to obtain the power from the interrogating radio frequency (RF) signal. On the other hand, passive chipless RFID tags have been proposed for enhancement of the backscattered signal in the detection of non-conductive pipes using commercial GPR systems. Both resonant tags and chipless tags in time-domain have been proposed for this purpose [5,17,18,19].

As the attenuation of the soil increases with the frequency, another critical decision is to select the frequency band of the tag. The patented system electronic marking system (EMS) by 3M [20] uses low-frequency tags that can yield great ground penetration depths. A similar solution is shown in the literature [21]. However, as frequency decreases, the minimum size of the tag increases considerably (due to antenna dimensions) [22], which makes this technique more expensive compared with conventional UHF RFID. The other option is to employ UHF passive RFID systems where higher transmitting power is allowed compared with Industrial, Scientific and Medical (ISM) bands. The exact frequency band varies between 865 MHz and 954 MHz depending on the region. In this frame, specific tag antenna design with a high level of gain for underground localization applications has been reported in the works of [23,24,25]. Recently, smart floor applications such as indoor mapping, localization, and robot guidance have been proposed in the literature [26,27,28,29] using different RFID bands.

The main drawback of these systems is that the continuous-wave (CW) signal powering up the passive tags is inevitably coupled to the receiver input as a strong self-interference, which presents a significant challenge to the reader’s receiver design. Moreover, a strong reflection due to the ground proximity is expected, and thus several cancellation techniques to overcome this problem in the RFID readers should be implemented [30,31].

The utilization of harmonic tags can be an interesting alternative that overcomes the undesired leakage between transmitter and receiver. This tag configuration consists of a frequency multiplier that generates a harmonic or sub-harmonic of the interrogation signal [32,33,34], providing a unique response signal independent of the leakage from the transmitter. Harmonic radars have been used to track insects for several years [32] using maritime radar-based technologies at X-band fundamental frequencies. The transponder typically consists of a dipole antenna directly matched to a Schottky diode [34]. In this application, it is possible to detect the insect at long distances within an environment with strong clutter, often using modified X-band marine radars transmitting high power. A commercial system to discover persons buried in avalanches is available from RECCO [35], which uses 917 MHz fundamental frequency to achieve outstanding ground penetration capability. Recently, several harmonic tags have been proposed in the literature for sensing applications [36,37,38] or combined with conventional UHF tags [39,40,41].

Previous works [42,43] discussed the feasibility of using harmonic tags for buried assets localization with operational frequencies of 2.5 GHz and 5 GHz, and measurements were realized using a high-dynamic range spectrum analyzer. However, a limited distance of interrogation was achieved (about 3 cm [42]). A humidity sensor based on a modulated tag has been presented by the authors of [38] with a read range of 7 m in free space. In the literature [44], modulated harmonic tags have been proposed for sensing applications where a read range of 4.5 m in free space is obtained using a non-linear transmission line as a frequency doubler. The main advantage of using the modulated harmonic tag compared with harmonic tags without modulation is the simplification in the receiver design as the modulated tag response at the second harmonic can be easily separated from the coupling at the second harmonic generated by the reader. Nevertheless, the modulated harmonic tags and chip-based RFID have the common drawback that the electronics used to modulate the harmonic components require a DC power source. In both cases, the tags [38,44] are powered based on an energy harvesting module, which converts the interrogation signal at UHF band to DC.

The main contribution of this work is to study the feasibility of using frequency-modulated harmonic tags for underground detection. To this end, an improved frequency-modulated harmonic tag is designed based on the work of [38]. A theoretical analysis is presented to estimate the read range, taking into account the different ground attenuations, which affect the interrogation signal and the second harmonic. The conversion loss of the frequency doubler is characterized according to the input power and is taken into account in the analysis. Measurements are performed for different types of soil (dry and wet), showing the possibility of detecting the tag buried at a depth of around 0.6 m, which highlights the potential of the proposed design. In contrast with simple one-bit harmonic tag configurations ([17,42,43]), where detection is based on the presence or absence of the tag, this work presents an advanced detection technique allowing the identification of the type of pipeline by specifying a different modulation frequency for each type. The study is completed with a proof-of-concept of a low-cost reader based on a simple Phase-Locked Loop (PLL) synthesizer used as a transmitter and an RTL software defined radio (SDR) used as a receiver. To the best of our knowledge, the frequency-modulated harmonic tag for localization of buried items is studied here for the first time.

The paper is structured as follows. Section 2 describes the system architecture and the harmonic tag design. Section 3 analyzes the tag performance based on the criterion of the maximum depth that can be detected depending on the attenuation of the ground. In this section, the theoretical reading range underground is estimated as a function of the soil composition and water content. A low-cost harmonic reader prototype based on an RTL SDR is presented in Section 4. A discussion of the results and a comparison with the state-of-art is given in Section 5. Finally, some conclusions are drawn in Section 6.

## 2. Harmonic Tag

### 2.1. Frequency Bands and Regulations

A passive harmonic tag is based on a non-linear device (usually a diode) that generates a useful emission at the second harmonic of the reader’s fundamental frequency. As in all passive RFID systems, higher transmitted power makes harmonic tag detection easier. The options for high power license-free operation are typically chosen between the 865/915 MHz, 2.4 GHz, or 5.8 GHz bands. In order to reduce losses due to the soil, the lowest frequency band is preferable. Therefore, the UHF RFID band at 865–868 MHz is chosen.

From a regulation point of view, one potential drawback is the interference of the second harmonic generated by the harmonic tag. The second harmonic (1730–1736 MHz in Europe or 1810–1830 MHz when 915 MHz is used) falls into the mobile Long Term Evolution (LTE) band 3. As the power received at the tag is relatively small, conversion loss is high. The result is that the second harmonic falls below the emission mask, as the typical license-free emission limit (FCC Part 15.209, in the United States) in which the effective isotropic radiated power (Equivalent Isotropic Radiated Power, EIRP) is −41.2 dBm at a distance of 3 m. In Europe, the ETSI standard EN 302 208 considers that the tag response is a spurious emission that must be below −47 dBm (Equivalent Radiated Power, ERP) at 1 m of distance. Additionally, for the United States, the FCC has determined that such tags (like RFID) are passive devices and the certification is done on the reader where the second harmonic can be easily filtered. Also, spread spectrum techniques used in modern mobile systems help to mitigate the effect of potential narrowband interference. Moreover, as the tag is interrogated from the reader using a very strong signal, typically −20 dBm or more to generate a useful return, any external interference over the reader transmitted signal would be negligible. It is also sometimes forgotten that conventional UHF tags work as non-linear elements, also generating spurious emissions [40,41], but fortunately, they do not interfere with the systems that operate in the mobile communications band due to their low level. This point will be discussed later according to the experimental results.

### 2.2. System Operation and Tag Description

**Figure 1:**
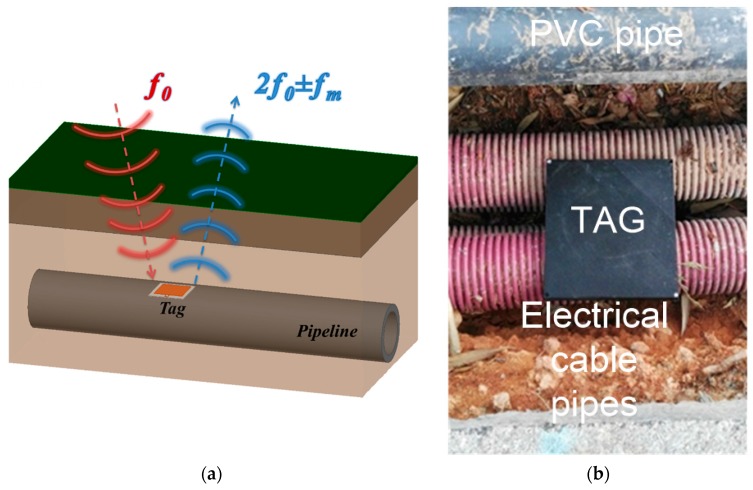
Block diagram of the proposed harmonic system (**a**) and an image of a typical installation (**b**).

The system is based on a harmonic reader configuration where the tag is placed over a pipeline as shown in Figure 1. The depth of the pipes of different services depends on the application, but the tag can be buried closer to the surface to facilitate its detection. This is an important difference compared with detection techniques based on GPR systems where the measured backscattered pulses correspond to the depth at which the pipes are buried. The reader interrogates the tag at the UHF frequency, *f*_0_ (a tone between 865 and 868 MHz). The tag is composed of a frequency doubler that generates the second harmonic at *2f*_0_, which is modulated (modulation frequency *f_m_*) and transmitted by an antenna tuned at the second harmonic. This modulated signal is then detected and filtered by the reader where undesired reflections at *f*_0_ coming from the ground or other objects (clutter) are eliminated. Hence, the harmonic frequency component (*2f*_0_ ± *f_m_*) allows for detecting the presence of the buried tag. In this work, the tag is modulated using a low-frequency oscillator for tag identification and facilitates its detection. The modulation frequency is adjusted depending on the asset or pipeline to be detected. Therefore, the measurement of the modulation frequency allows for identification of the tag. In addition, the introduction of the tag modulation concept simplifies the design of the reader as the high isolation between the fundamental frequency and second harmonic is not required.

**Figure 2:**
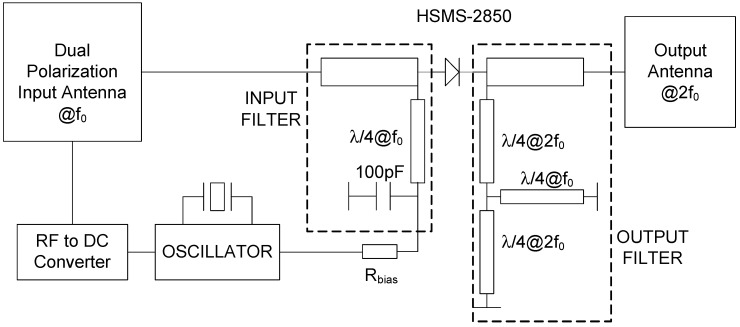
Block diagram of the tag designed.

**Figure 3:**
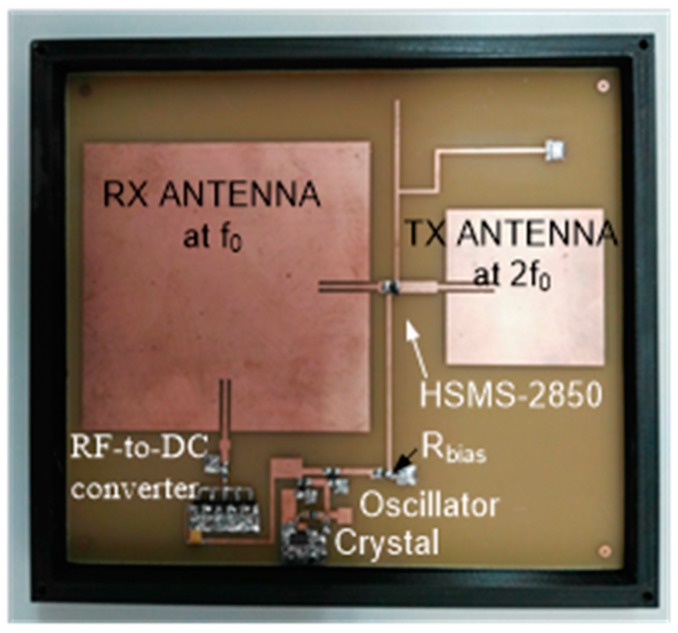
Photography of the implemented tag.

**Figure 4:**
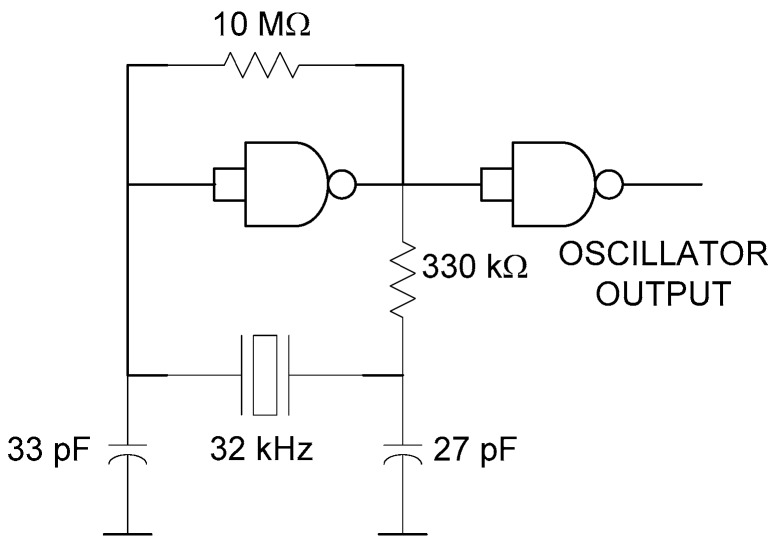
Schematic of the quartz crystal low-power oscillator.

The tag is inspired from a previous harmonic tag used as a humidity sensor [38], but with some modifications that have been performed to stabilize the modulation frequency, and the antennas have been redesigned for the underground operation. The block diagram of the proposed tag is shown in Figure 2. A prototype of the tag is implemented on standard 1.6-mm thick FR4 substrate, which is shown in Figure 3. The reader transmits a continous-wave (CW) signal at frequency *f*_0_ using a circularly polarized antenna. The tag receives this signal using a dual-polarized patch antenna tuned at *f*_0_. One of the outputs of this antenna is connected to the frequency doubler and a linear-polarized transmitter antenna at 2*f*_0_, and the other output to an RF-to-DC converter. The converter’s output is used to bias a low voltage oscillator that modulates the tag response by changing the bias of the Schottky diode at the frequency doubler. To this end, an oscillator based on two low-power NAND gates (74AUP1G00, Plano, TX, USA) and a quartz crystal at the modulation frequency (32 KHz) are used in this work. The high-quality factor of the quartz resonator allows using low-resolution bandwidth filters (in the receiver) that reduce the noise and make the tag detection easier, because the oscillation frequency is constant independently of the harvested voltage in contrast with the previous tag [21]. The schematic of the oscillator is shown in Figure 4. The frequency doubler is based on a zero-bias Schottky diode (Avago Technologies, San Jose, CA, USA, model HSMS-2850) [21]. The fundamental signal at *f*_0_ is filtered using a high-pass filter (output filter in Figure 2) implemented with a λ/4 stub at 2*f*_0_. The second harmonic is blocked at the frequency doubler input using a short-circuited λ/4 stub at *f*_0_ and a bypass capacitor. The output of the frequency doubler can be modulated by changing the diode DC bias point. To this end, a bias resistor (R_bias_) is inserted at the output of the oscillator. The quarter wavelength line end is connected to the ground at RF using a bypass capacitor. The high value of the resistor (1 kΩ) also blocks the RF signal and limits the DC current consumption, while the DC return is allowed through the resistor.

### 2.3. Tag Antenna Design

The antennas have been designed using the Keysight Momentum simulator. The size of the receiver patch is 75 mm by 75 mm, and a 1-mm wide coplanar line is employed to match the antenna. The length of the coplanar line is 14 mm. The transmitter patch has a dimension of 40.5 by 40.5 mm, and the inset of the 1-mm wide coplanar line has a length of 12 mm.

**Figure 5:**
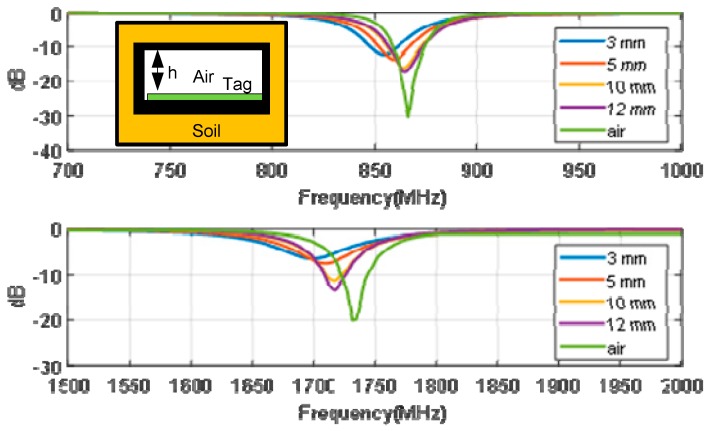
Reflection coefficient of the patch antenna at the fundamental frequency (**top**) and the second harmonic (**bottom**) as a function of frequency for different spacer thickness (h = 3 mm, 5 mm, 10 mm, 12 mm, and the tag in the air). The inset figure shows a schema of the cross-section of the box in the soil.

Patch antennas are narrow-band antennas, especially if thin substrates are used [45]. As the tag is buried in soil, a detuning due to the presence of high-permittivity material can be expected. In addition, the tag must be protected from contact with moisture through a plastic box (see Figure 3). By means of electromagnetic simulations, the effect of the height of the box and the soil permittivity is studied. Figure 5 shows the reflection coefficient of the antennas for different air spacers (placed between tag and soil due to the height of the protection box) varying between 3 mm and 12 mm. A relative permittivity of 7 and loss tangent of 0.25 is considered as a worst case in the simulations in order to simulate wet soil [46,47]. The plastic box is made with polylactic acid (PLA) using a 3D printer. A 1-mm thick top cover has been considered in the simulations and in the prototype. The dielectric permittivity (ε_r_ = 2.8 and tanδ = 0.003) used in the simulations is taken from the work of [48]. It is shown that for a spacing of 12 mm, the detuning due to the presence of soil is small. Consequently, in order to avoid the antenna’s detuning, the tag is inserted into a plastic box with an air gap of 12 mm between the antenna and the soil, which also protects the tag against water (see Figure 3).

### 2.4. Tag Characterization

**Figure 6:**
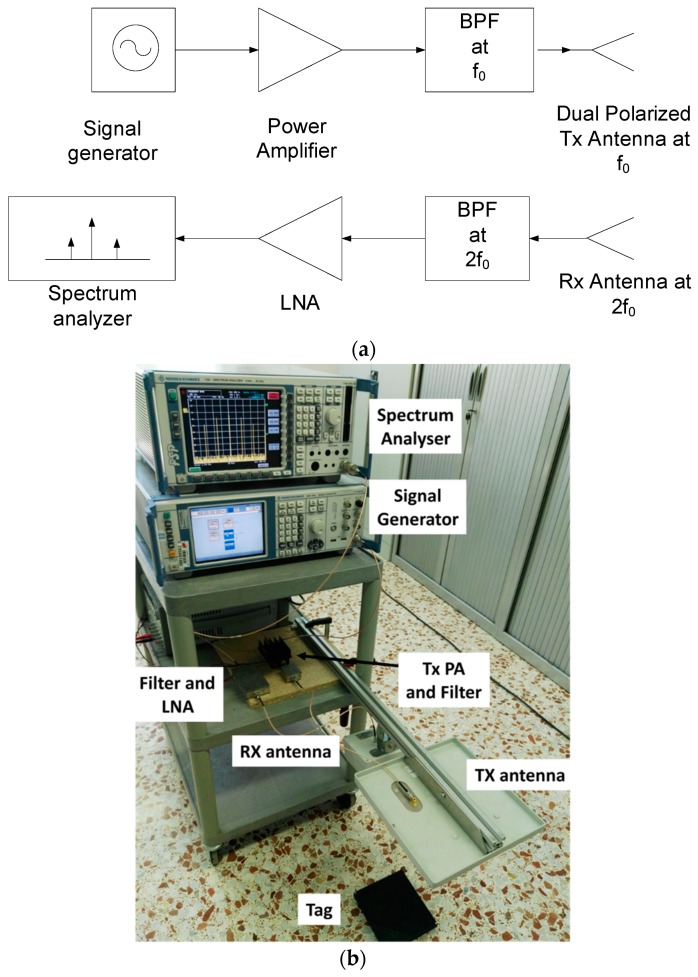
(**a**) Block diagram of the experimental setup based on laboratory instrumentation and (**b**) photograph.

A reader based on laboratory instruments to characterize the tag has been designed. A block diagram of the experimental setup and a photograph are shown in Figure 6. It is composed of a signal generator (Rohde & Schwarz SMA100A, Moorpark, CA, USA) connected to a power amplifier (PA, Minicircuits ZHL-3010, New York, NY, USA). It is tuned at the fundamental frequency (*f*_0_) and is capable of performing sweeps in frequency and varying its power level. A commercial circular-polarized UHF RFID antenna (Feig) is used in transmission. The receiver is a spectrum analyzer (Rohde & Schwarz FSP30, Cary, NC, USA) with a preamplifier and a Band-Pass Filter (BPF) tuned at the second harmonic band to avoid the saturation of the low-noise amplifier (LNA). A linear-polarized antenna (Geozondas AU-1.0G4.5GR, Vilnius, lithuania) is used in reception. In addition, a multimeter is connected to the output of the RF-to-DC converter to measure the rectified voltage. An oscilloscope is used to measure the oscillation frequency and to check the oscillation of the tag.

**Figure 7:**
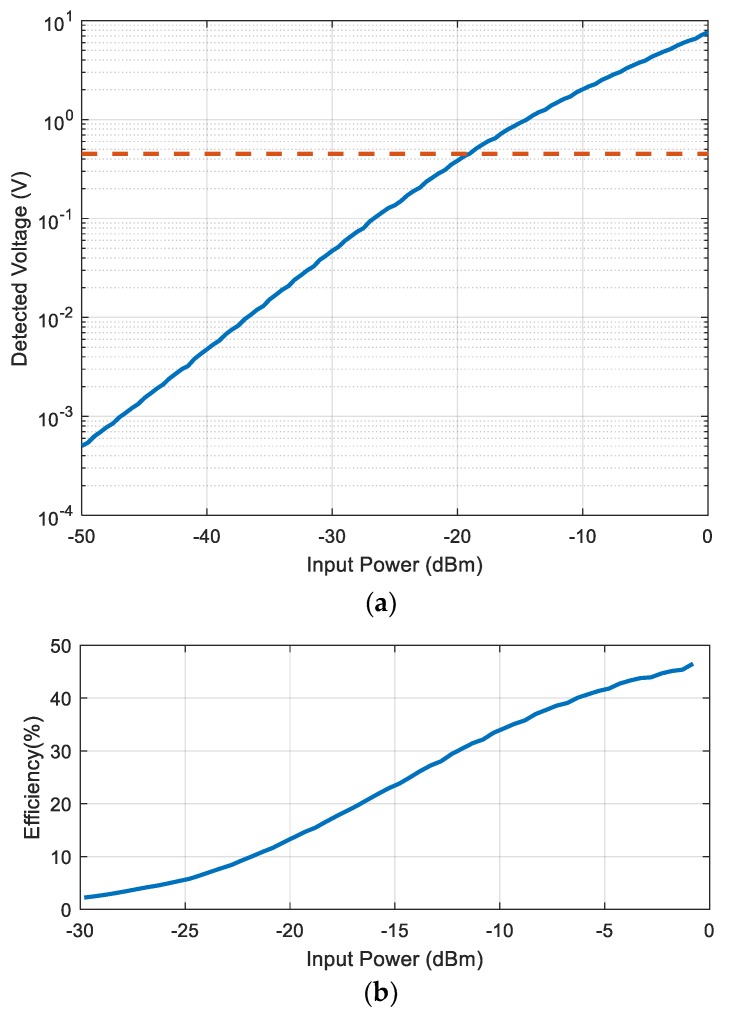
(**a**) Measured detected voltage as a function of input power at 868 MHz. The dashed line shows the minimum voltage needed to power up the oscillator (0.45 V). (**b**) Measured efficiency as a function of the input power at 868 MHz.

Compared with harmonic tags based on simple diodes [32,33], the oscillator requires a power source that is obtained from the input rectenna. The RF-to-DC converter is based on a diode voltage multiplier using five stages of series-connected zero bias diodes (Avago Technologies, model HSMS-2852) [38]. The matching network at the input of the circuit is optimized to be matched between −20 dBm and −10 dBm. It consists of an LC matching network and it is tuned with the network analyzer. This circuit needs only 0.45 V DC to oscillate. From the characterization of the RF-to-DC converter that is shown in Figure 7, a threshold power of around −20 dBm is required to bias the circuit. At this threshold power, the efficiency obtained with the oscillator connected to the output is about 15% (Figure 7b).

**Figure 8:**
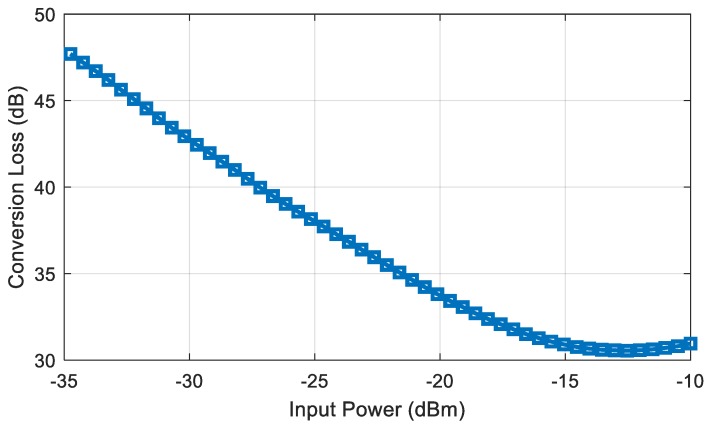
Measured conversion loss as a function of input power when the frequency doubler is modulated with a 32 KHz 0.45 V square wave.

The tag read range will be studied in the next section. To this end, the conversion loss of the doubler must be known. When the tag is modulated with an ideal square wave train, the expected spectrum is the Fourier transform of the waveform pulse at the harmonics of the modulating frequency (*f_m_*). Therefore, there is a modulation loss (K) with respect to the unmodulated case [49]. A prototype of doubler for characterization purposes has been manufactured. Figure 8 shows the measured conversion loss when the doubler is externally modulated with a 32 KHz square wave of 0.45 V (minimum DC voltage needed by the oscillator). The conversion loss is obtained from the difference between the measured power at the output at 2*f*_0_ + *f_m_* and the input power at *f*_0_, obtaining a value around 34 dB for −20 dBm of input power.

**Figure 9:**
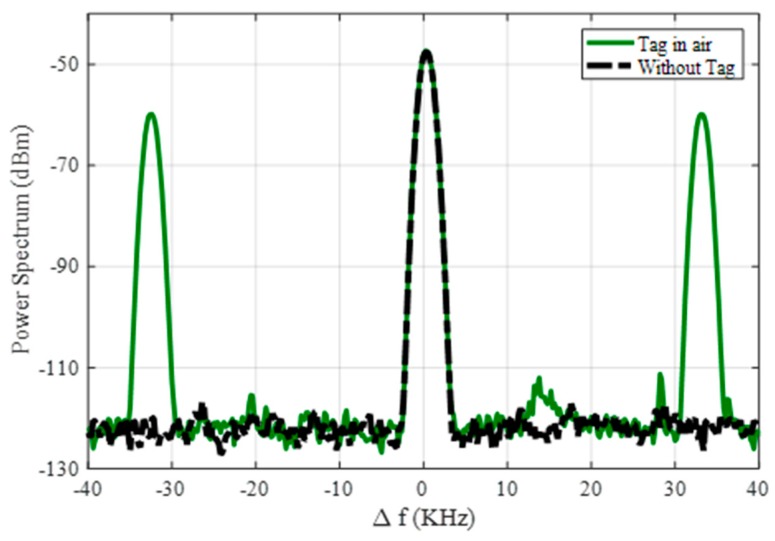
Measured spectrum at 2*f*_0_ = 1732 MHz (∆*f* = 0) for two scenarios: without the tag and with the tag in the air at 0.7 m from the TX/RX antennas.

**Figure 10:**
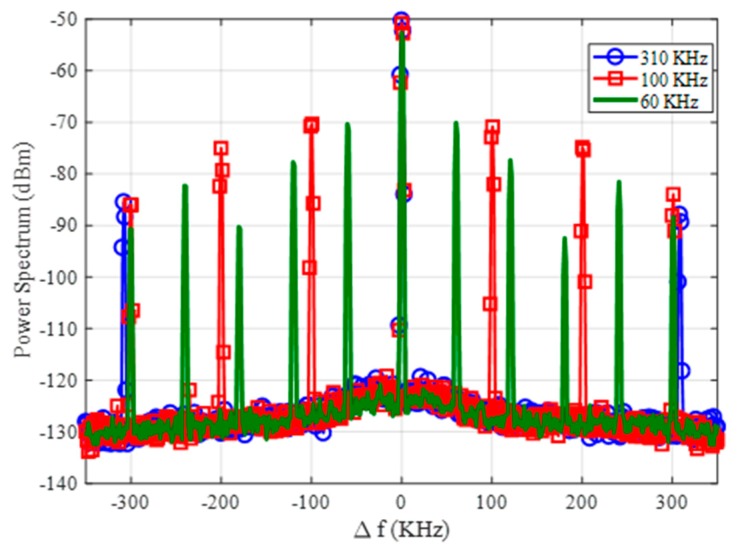
Measured spectrum at *2f*_0_ = 1732 MHz (∆*f* = 0) using three different modulation frequencies.

Compared with the tag presented in the work of [38], the modulation frequency is determined by the quartz crystal, and it is not sensible to the variation of the bias voltage. Therefore, as the power reaching the tag overcomes the oscillator power threshold, the modulation frequency detected does not vary with the input power. The frequency modulation of the tag proposed in this work has several advantages. First, it overcomes the inevitable parasitic coupling between transmitter and receiver at the second harmonic. This undesired harmonic signal is mainly generated by the output power amplifier in the reader transmitter. Figure 9 shows a couple of measurements of the receiver spectrum using the setup of Figure 6 considering the tag over the ground located at 70 cm from the reader antennas and without the tag. As shown in Figure 9, a residual level at *2f*_0_ is always detected, despite the use of filters, even in the absence of the tag. Therefore, this modulation technique increases the tag read range because measurements are performed at *2f*_0_ + *f_m_*, and thus are not affected by the coupling limitations. Furthermore, different oscillation frequencies can be chosen, as shown in Figure 10, by changing the quartz crystal depending on the target to be identified (e.g., water, gas, or electricity installations). These features highlight the potential of applying the proposed design in this work. The received level clearly complies with the regulations.

## 3. Read Range

### 3.1. Soil Attenuation Model

In order to estimate the maximum underground range for tag detection, a study of the soil’s attenuation loss is required. The attenuation can be obtained from the complex dielectric constant of the soil. It can be divided into two parts. The first one, called the reflection attenuation, is the result of the reflection produced when the wave changes medium (air–soil), and the second one is the attenuation due to the soil’s loss. These components can be estimated from the dielectric constant of the soil. The reflection attenuation (*L_r_*) is independent of the distance, and can be calculated for normal incidence using the Fresnel coefficient.

(1) $$L_{r}(dB)=-10\log\left(1-{\left|\frac{\sqrt{\varepsilon_{s}}-1}{\sqrt{\varepsilon_{s}}+1}\right|}^{2}\right)$$

The attenuation loss due to the propagation (*L_p_*) can be calculated from the complex propagation constant γ and the propagation distance *d*.

(2) $$L_{p}(dB)=8.686\alpha (Np/m)\times d$$

(3) α(Np/m)=−Im(γ)=2πfcεs′2(1+(εs″εs′)2−1)

where $\varepsilon_{s}=\varepsilon_{s}\prime -j\varepsilon_{s}\prime \prime$ is the complex dielectric relative constant of the soil and *c* is the speed of light in the vacuum.

The dielectric constant depends on soil physical properties including bulk density, soil texture, and the level of water content. The dielectric soil properties can be measured following the procedure described in the literature [43] from the measurement of the S parameters with an especial coaxial fixture. After de-embedding the S parameter of the coaxial line with the soil as a dielectric, the dielectric constant can be found using the Nicholson and Ross algorithm. In this work, we use the model of Peplinski et al. [46,47], which is validated for the frequency range of 0.3–1.3 GHz. This model provides expressions for soil dielectric constant as a function of the soil volumetric water content θ_V_, the frequency *f*, the fraction of sand particles *S*, the fraction of clay particles *C*, the density of the soil particles ρ_S_ (a typical value is 2.66 g/cm^3^), and the bulk density of the soil ρ_B_ (an average value is 1.6 g/cm^3^).

**Figure 11:**
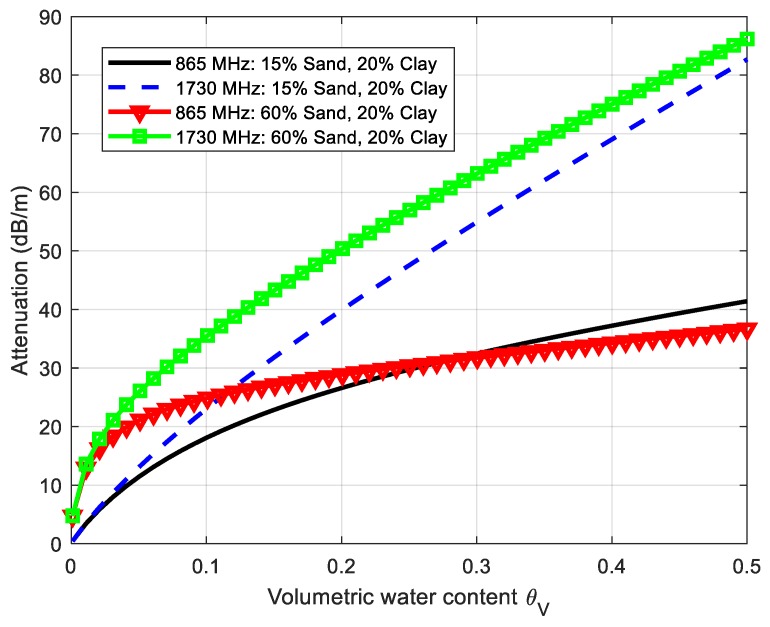
Simulated attenuation coefficient in dB/m calculated from Equations (2) and (3) as a function of the volumetric water content of the soil at 865 MHz and 1730 MHz for two soil cases.

**Figure 12:**
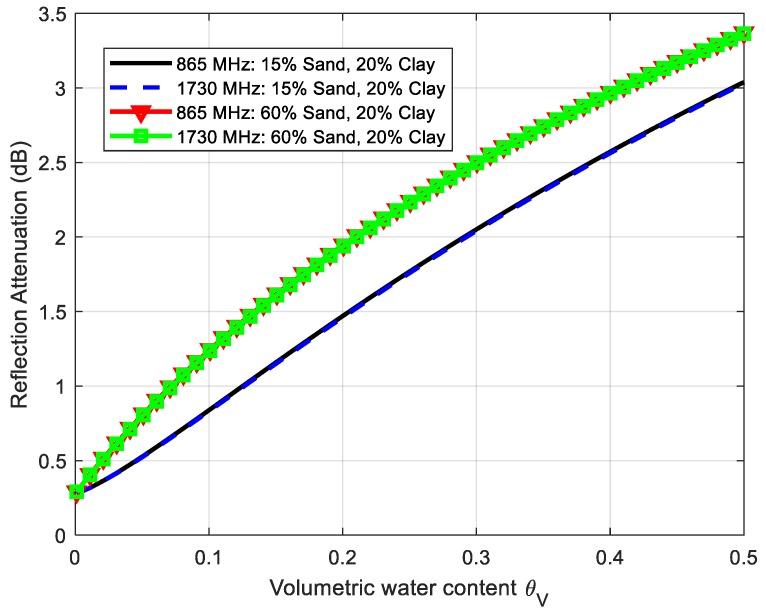
Simulated reflection attenuation in dB calculated from Equation (1) as a function of the volumetric water content of the soil at 865 MHz and 1730 MHz for two soil cases.

Using Equations (1)–(3) and the dielectric constant given by Peplinski’s model, Figure 11 and Figure 12 depict the simulations of the attenuation coefficient α in (dB/m) and the reflection loss at *f*_0_ (865 MHz) and 2*f*_0_ (1730 MHz) for two soil cases; namely, case 1 (sand fraction S = 15%, clay fraction C = 20%) and case 2 (sand fraction S = 60%, clay fraction C = 20%). The attenuation coefficient increases with the frequency especially when the water content increases, and it is noticeably higher at the second harmonic. These simulations show that the attenuation can vary considerably depending on the composition of the soil. On the other hand, the loss due to signal reflection at the boundaries of the two mediums (air and soil) is considerably lower than the attenuation due to propagation.

### 3.2. Link Budget

The read range in an RFID system can be limited by the uplink (reader to tag) or by the downlink (tag to reader) [49,50]. In the uplink, the tag must receive enough power to be activated (*P_r, tag_* > *P_th_*), whereas in the downlink, the backscattered power (*P_r, reader_*) by the tag must be higher than the reader sensitivity (*S_min_*).

In a conventional UHF RFID system, as the reader’s antenna interrogates the tag in the air, the main problem is the fading due to multipath interference from objects, especially in indoor environments. The read range is limited by the tag sensitivity as the propagation loss is the same in both directions.

In this application, the reader antennas are close to the ground (for example, at 10 cm in order to avoid detuning by the proximity of the ground), and the main source of attenuation is the result of the loss of the soil that depends on its composition. Therefore, the read range is the maximum distance that complies with the two conditions (*P_r, tag_ > P_th_* and *P_r, reader_ > S_min_*). The main advantage of harmonic systems is that the receiver sensitivity in the reader may be noticeably better than in a conventional reader because the coupling interference between the reader transmitter and receiver can be filtered as the receiver is tuned at the second harmonic. As discussed previously in Section 2, the effect of residual coupling at 2*f*_0_ can be reduced by improving the filters in the transmitter and receiver or by modulating the radar cross-section as in conventional tags. In this work, the second option is investigated.

The power received at the tag from the reader can be calculated from the Friis equation, the reflection loss, and the attenuation [38], as follows:

(4) $$P_{r,tag}\left(dBm\right)=EIRP\left(dBm\right)-10log\left(4\pi d_{g}^{2}\right)+G_{tag}\left(dB\right)+10log\left(\frac{\lambda^{2}}{4\pi }\right)-L_{r}\left(dB\right)-L_{p}\left(d\right)\left(dB\right)$$

where *d_g_* is the distance from the reader antenna to the ground; *λ* is the wavelength at *f*_0_; EIRP is the equivalent isotropic radiated power of the reader (limited by the regulations); *G_tag_* is the gain of the tag antenna; *L_r_* and *L_p_* are the reflection and propagation loss of the soil in dB, given by (1) and (3), respectively; *L_p_* is a function of the underground distance; *d*. *G_tag_* is the gain of the tag antenna; and *G_R, reader_* is the gain of the reader antenna in reception.

The backscattered power in a harmonic tag is given by the following [38]:

(5) $$P_{r,reader}\left(dBm\right)=P_{r,tag}\left(dBm\right)-CL\left(dB\right)+G_{tag,2}\left(dB\right)-10log\left(\frac{\lambda_{2}^{2}}{4\pi }\right)+G_{R,reader}\left(dB\right)$$

where *CL* is the doubler conversion loss (including the modulation factor, that is a function of the *P_r, tag_* power and is given in Figure 8), *G_tag_, *_2_ is the antenna gain at 2*f*_0_, and *λ*_2_ is the wavelength at 2*f*_0_ (=*λ*/2).

### 3.3. Tag Read Range

**Figure 13:**
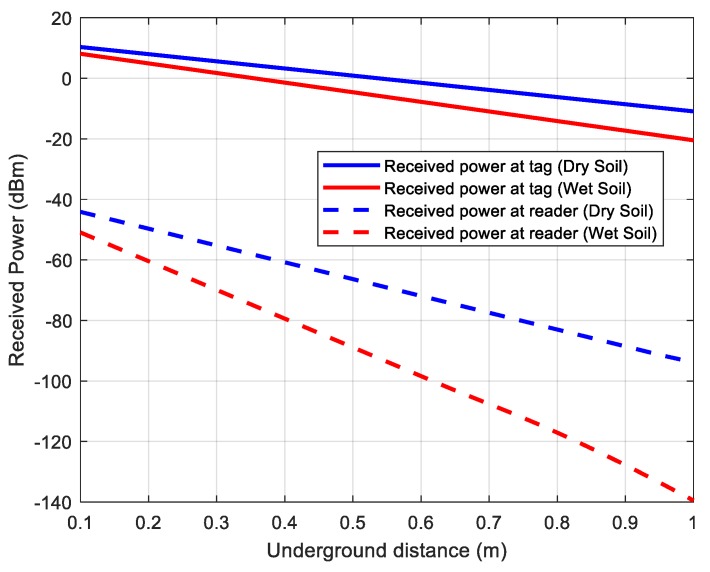
Simulated received power at tag and reader for dry (solid lines) and wet soil (dashed lines).

Figure 13 shows read range simulations as a function of the received power for dry and wet soil (case 2: 60% sand, 20% clay). The volumetric water content (VWC) in the case of wet soil is considered the worst case, close to the saturation (30%). Considering tags with a threshold power of −20 dBm, the link budget is limited by the downlink (received power at the reader). Table 1 summarizes the main parameters used in calculations.

These results show that the read range underground depends on the water content. In the case of wet soil, the read range is about 0.7 m for a receiver sensitivity of −110 dBm (considering a 10 dB of margin over the noise floor). Indeed, it is different than traditional UHF RFID applications in the air, where the read range is limited by the threshold tag power.

Although the attenuation of the soil at the second harmonic is higher than at the fundamental frequency, better read range performance is obtained for the harmonic tags because the receiver sensitivity is better than that of conventional UHF readers (≈−73 dBm).

**Figure 14:**
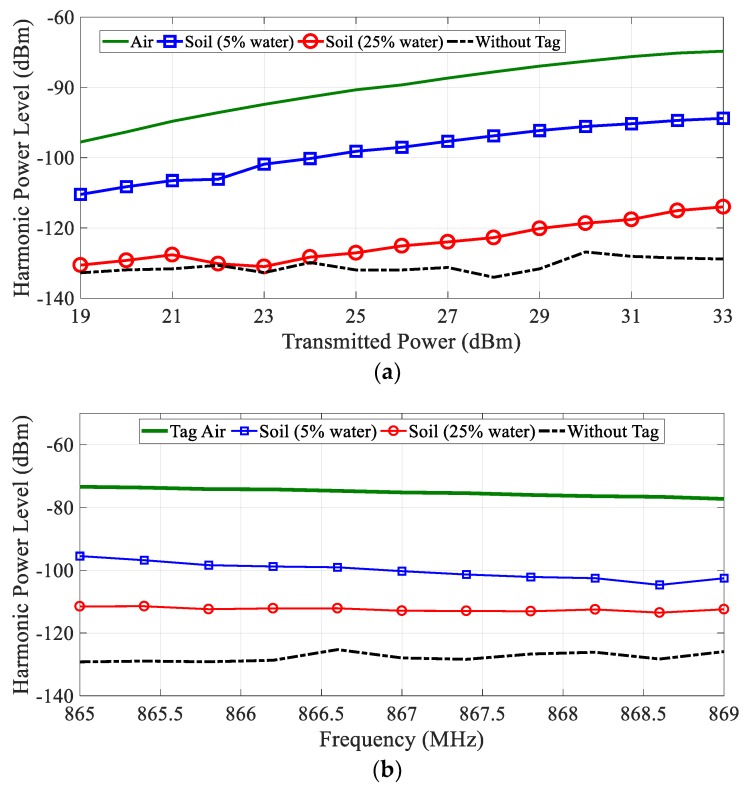
(**a**) Measured tag response under different conditions (tag in the air, buried in the soil, and without tag) as a function of the transmitted power (equivalent isotropic radiated power—EIRP) and a function of the input frequency (**b**) for the EIRP of 33 dBm.

Measurements have been performed with the instruments shown in Figure 6 and the tag buried underground. To this end, the tag was placed in a cylindrical plastic container with a diameter of 0.6 m filled with soil with known water content. This container was placed inside a hole in the ground. The volumetric water content (VWC) is determined from the ratio between the soil and water introduced in the container. The antennas of the reader were placed 10 cm away from the ground. Figure 14 shows measurements of the received power (at frequency *f*_0_ *+ f_m_*) with the tag buried 60 cm underground for two volumetric water contents (5% and 25%). The first water content is close to the residual value of dry soil and the second is close to the saturation value for the soil used. This figure also compares a measurement of the tag in the air at the same distance from the antennas (70 cm) and the measurement of the noise floor (measurement without tag). The equivalent isotropic radiated power (EIRP) is swept from 19 dBm to 33 dBm, as shown in Figure 7a, and the frequency is swept in the European RFID band (865–869 MHz) for the case of EIRP of 33 dBm. The measured power level with the spectrum analyzer is referred to the antenna input after subtracting the gain of the chain of amplifiers and filters connected to the input of the spectrum analyzer to reduce the noise factor of the instrument. The effect of the attenuation of the soil can be observed, especially for high volumetric water content. An attenuation of about 19 dB (or 32 dB/m) and 25 dB (or 42 dB/m) between the case of the tag and the air and the tag buried for 5% and 25% VWC, respectively, is found. These values of attenuation are within the order of magnitude obtained in Figure 11. A difference of about 10 dB over the noise floor for the case of 25% of VWC is observed, which leaves a certain margin to detect the tag if there are variations in attenuation caused by changes in the composition of the soil. A nearly flat response is obtained in all frequency bands (Figure 14b).

## 4. Low-Cost Harmonic Reader

**Figure 15:**
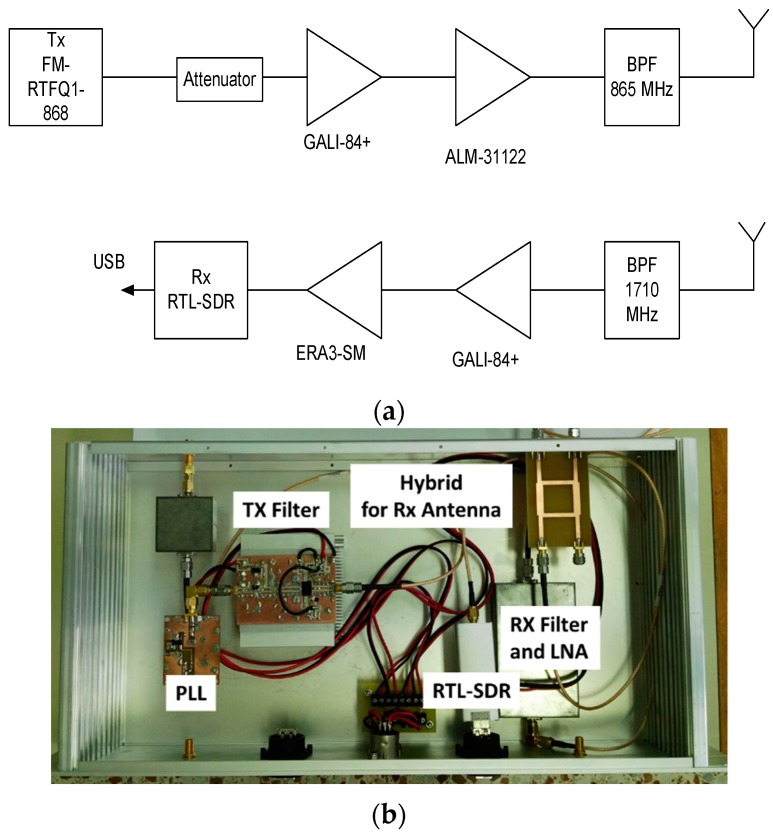
Block diagram (**a**) and photography (**b**) of the low-cost reader implemented.

**Figure 16:**
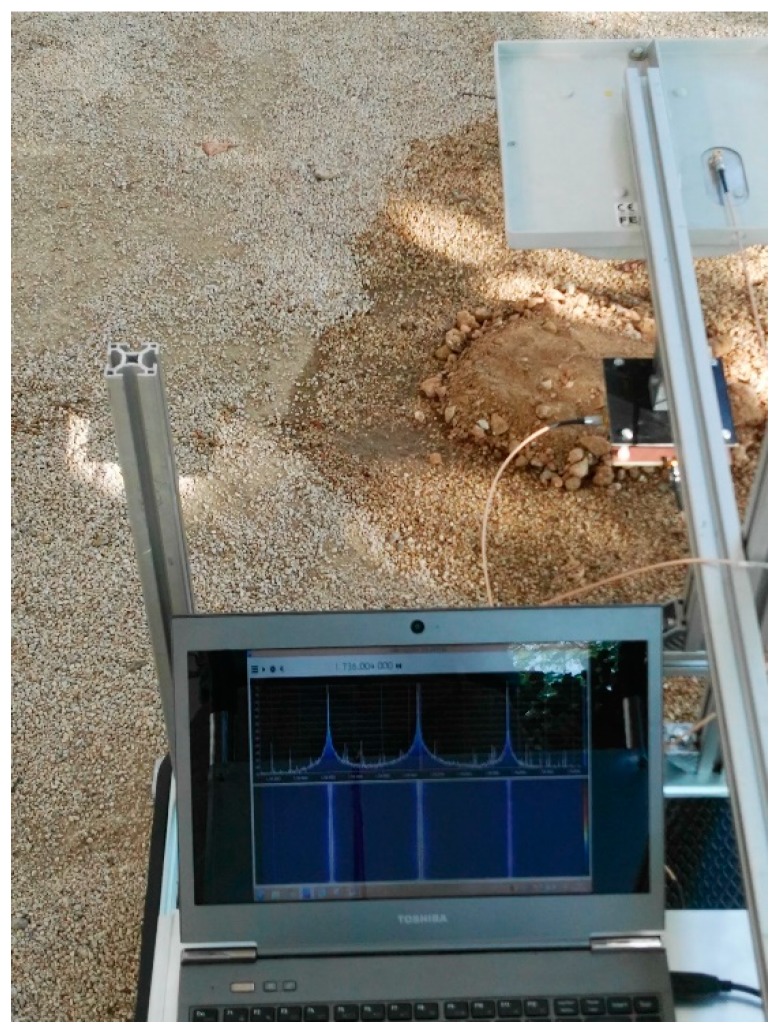
Photography of the system and measurement setup.

**Figure 17:**
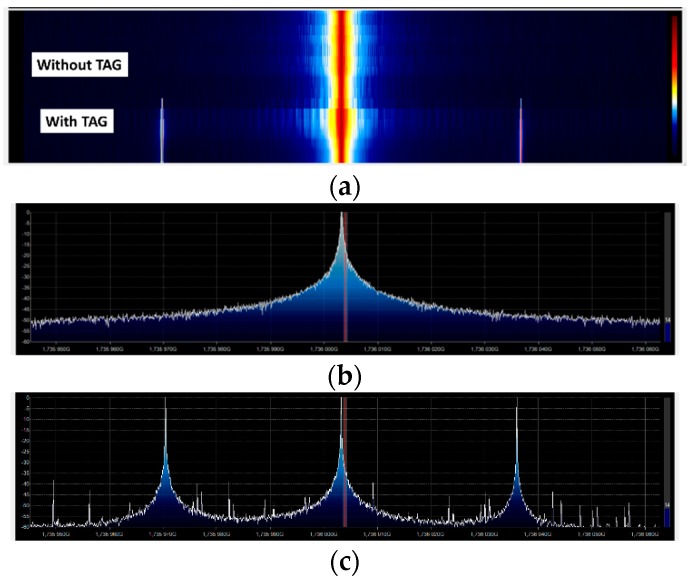
Screenshot of measurements with SDR# software: (**a**) spectrogram without the tag and with the tag, (**b**) cut of the spectrum without the tag, (**c**) spectrum with the tag.

Unfortunately, harmonic readers are not yet commercially available. A proof-of-concept reader based on a generator with a power amplifier and a spectrum analyzer is implemented. A low-cost implementation based on an SDR device (RTL–SDR based on chipset R820T2 RTL2832U) and free open source SDR software (SDRsharp) is presented in Figure 15. A photograph of the system is shown in Figure 15b. The transmitter is based on a PLL synthesizer where a low-cost 868 MHz ISM band FM transmitter (FM-RTFQ1-868) from Telecontrolli is employed. A chain of amplifiers composed by Minicircuit Gali-84+ and Broadcom ALM-31122 are used to reach maximum output power (26 dBm). An attenuator is included to avoid the saturation of the amplifiers and to control the output power. Custom combline passband filters were designed using microstrip technology. A bandpass filter centered at 868 MHz is added to filter spurious emissions at the second harmonic. The receiver is composed of a bandpass filter at the second harmonic and two amplifiers. The first amplifier (Minicircuits GALI-84+) presents a high compression point to avoid the saturation of the chain. The second block amplifier is the Minicircuits ERA-3SM. The microstrip combline filters are designed to be connected both before and after the transmitter/receiver antenna. A dual-polarized RFID antenna (model FEIG gain 8.5 dBi) is used in transmission, as well as a custom circular-polarized patch antenna (a circular path with a hybrid) with gain 6 dBi in reception. A photograph of the system mounted on a trolley is shown in Figure 16. In order to control the soil’s water content, the tag is submerged in a plastic container within a hole in the ground. The water content of the soil of the container is known. The tag is placed again at a distance of 70 cm from the TX/RX antennas and buried 60 cm under the ground. Figure 17 shows a screenshot of the SDRsharp corresponding to the measurement of the tag’s response when the prototype of the reader passes over it. When the tag is out of range, a central peak caused by the parasitic coupling appears in the center of the spectrum (Figure 17b), whereas when the antennas are on top of the tag, a couple of peaks at the modulation frequency arise around the central frequency (Figure 17c). A version of SDRsharp is available also for Android devices; therefore, the software can run on a small tablet or smartphone in order to reduce the weight of the system in a future potential commercial product.

**Figure 18:**
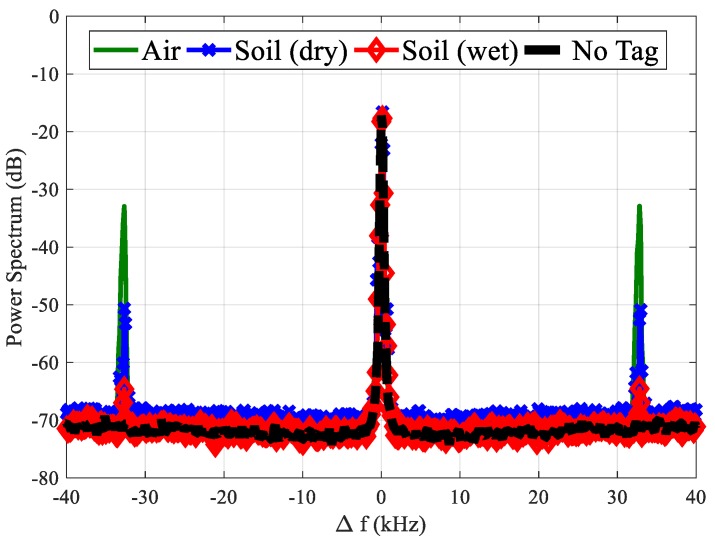
Comparison of tag measured spectrum response at 2*f*_0_ = 1732 MHz as a function of the offset frequency (∆*f*) for three configurations: (i) tag in the air, (ii) tag buried under dry soil, and (iii) tag buried under the wet soil (VWC 15%).

Finally, Figure 18 shows a comparison between the tag responses using the proposed low-cost reader for three configurations. The comparisons between previous measurements in Figure 18 demonstrate the limitation of this reader because the PLL presents a higher noise level. This affects the measurements performed under wet soil because the tag cannot be detected when the water content is higher than 15%.

## 5. Discussion

An estimation of the cost of the system (for medium–large quantities) is given in Table 2. The price of the tablet, smartphone, or PC for data acquisition has not been included, because the software (i.e., SDRsharp) can run on different platforms and does not have specific requirements. The cost of the reader is around two hundred dollars and the tag is about four dollars. The cost of the reader is significantly lower than a UHF reader (between $1000 and $2000). The cost of a tag is comparable to a UHF tag for metallic applications, however, specific Integrated Circuit (IC) should be designed in order to improve the integration and reduce cost.

In order to compare the proposed solution with the state-of-art, Table 3 compares several techniques proposed in the literature for buried utility localization. The proposed system is easy to use and the cost is significantly lower than that of GPR systems used for pipe localization that, in addition, require specialized operators to interpret the measurements. In order to improve the resolution, GPR systems for pipe detection commonly use antennas between 900 MHz and 2000 MHz, therefore, the detection depth depends on soil losses and water content as in the proposed system. An enhancement system based on resonators has been proposed in the literature [5], obtaining a detection depth of 0.6 m at around 900 MHz. Enhancement techniques for improving the detection of GPR signals based on antennas loaded with delay lines are proposed in the works of [17,18], obtaining detection depths of 1.1–1.2 m in dry soils. Recently, surface acoustic wave (SAW) RFID tags at 915 MHz have been proposed in the literature [51], obtaining a detection depth of 1.3 m. In the work of [24], UHF RFID metal tags are used for manhole detection. The highest depth underground that is detected is 10 cm. Also, depths of about 10 cm are reached for tags embedded in bricks and mortar in the work of [29]. Special antenna designs for buried tags using air spacers as proposed iin the work of [23], or antenna designs used in this work can reduce the detuning effect due to the soil. However, the higher receiver sensitivity in the harmonic system allows the detection at higher depths compared with UHF systems. Few experimental results have been found in the literature employing buried harmonic tags. Experimental results with a harmonic tag buried at 3 cm under the sand are given in the work of [42], transmitting 7 dBm at a 2.4 GHz ISM band. In the work of [17], a harmonic tag based on silicon PN diode at 150 MHz is proposed for detection of buried object, but only experiments in free space are presented. Harmonic tags based on a varactor diode at 400 MHz have been proposed for the underground detection in the work of [43], but again experimental results in free space have only been presented up to 76 cm. Several low-frequency (LF) RFID systems (125–135 kHz) have been investigated for the underground detection in the work of [52,53]. However, their detection depths are often far from the 1.5 m obtained with the commercial system of 3M [20] at LF. This system exploits the noticeably less attenuation at LF compared with that at UHF or microwave systems to achieve this depth. In order to obtain higher detection depths or detection under ice, an active transponder must be used [14]. The main drawback of an active transponder is the finite lifetime of the batteries and the cost of the tag compared with passive RFID tags. Another potential application of the proposed sensor is smart floor applications for indoor mapping, localization, and guidance [29]. Both UHF [29] and High Frequency (HF) RFID [28] are often proposed for smart floor applications. However, in the work of [27], passive reflectors buried under the tiles and semipassive ultra-wide-band (UWB) tags are detected with a low-power Impulse Radio-UWB (IR-UWB) radar using a time-domain analysis similar to the one used in GPR systems. In smart floor applications, the tag is often under tiles in indoor environments and the depth of the buried tag is from a few millimeters to 1 cm. Therefore, the attenuation introduced by these materials is lower than that due to tags underground, especially if the soil is not dry.

## 6. Conclusions

A passive harmonic tag has been presented for buried assets localization. In order to identify different utility services underground and facilitate the detection, the tag is modulated using a low-power oscillator that is fed from RF energy harvesting. The theoretical read range is estimated from the attenuation model found in the literature. The experimental results show that the harmonic tag reaches read ranges up to 60 cm under the soil with a 25% water content. A low-cost harmonic reader prototype based on an inexpensive SDR receiver was also developed.

## Figures and Tables

**Table 1:** Parameters used in the read range simulations. EIRP—equivalent isotropic radiated power; VWC—volumetric water content.

Parameter	Value	Unit
Transmitter EIRP	2	W
Frequency *f*_0_	865	MHz
Tag antenna gain, *G_tag_* at *f*_0_	6	dB
Tag antenna gain, *G_tag_, *_2_ at 2*f*_0_	6	dB
Reader antenna gain, *G_R_*	6	dB
Volumetric water content VWC for the wet soil	30	%
Distance to ground, *d_g_*	10	cm

**Table 2:** Estimated cost of different parts of the system. SDR—software defined radio.

Parameter	Cost ($)
RTL–SDR	20
Reader Antennas	100
Filters	10
Amplifiers	10
Power Supply	20
Connectors and box	20
Trolley	50
Tag	4

**Table 3:** Comparison of different techniques for detecting underground objects. GPR—ground penetrating radar.

Reference	Technique	Deep Underground	Comments
[5]	GPR (time domain)	0.6 m	Detection of pipes using tag resonators with GPR (GSSI model) at 900 MHz with tag resonators.
[17]	GPR (time domain)	1.1	Detection of a dipole antenna with a delay line buried using GPR (Sensors model) operating at a center frequency of 900 MHz in a dry sand.
[18]	GPR (time domain)	1.2	Detection of FAT dipoles with delay lines at 900 MHz with VNA in dry soil.
[19]	GPR (time domain)	0.45 m	Detection of resonant tags with GPR (GEOTCH OKO-3 model) at 700 MHz in dry sand
[51]	SAW RFID 915 MHz	1.3 m	SAW RFID at 443 MHz and 915 MHz in dry soil transmitting 30 dBm.
[20]	LF RFID	1.5 m	Electronic marking system (EMS) by 3M.
[52]	LF RFID (134.2 kHz)	0.24 m	Passive integrated transponder (PIT) for tracking soil movements.
[53]	LF RFID (135 kHz)	NA	Underground pipeline location.
[28]	HF RFID	8 mm	Smart floor application with standard 13.56 MHz tags under the tiles.
[24]	UHF RFID	10 cm	UHF RFID metal tags are used for manhole detection.
[29]	UHF RFID	10 cm	Metal UHF tags embedded in bricks and mortar.
[27]	IR UWB radar	1 cm	Detection of metallic reflectors under the floor for smart floor application with UWB radar.
[42]	Harmonic tag at 2.4 GHz	3 cm	Harmonic tag with a reader transmitting 7 dBm.
[43]	Harmonic tag at 400 MHz	NA	Experimental results in free space up to 0.76 m at 400 MHz using a varactor based harmonic tag.
[17]	Harmonic tag at 150 MHz	NA	Harmonic tag at 150 MHz (preliminary experiments in free space).
This work	Harmonic UHF	30–60 cm	Harmonic tag with harvester and modulator.
[14]	Active tag at 315 MHz	NA	500 mW transmitter under the ice.
